# Supplementary material for: A zero-cost attention-based approach to promote cleaner streets: A Signal Detection Theory approach in Parisian streets
Source: PLoS One. 2023 Apr 26;18(4):e0284272. doi: 10.1371/journal.pone.0284272 (PMC10132669; doi:10.1371/journal.pone.0284272)
Supplement: S1 Material — (DOCX) [file pone.0284272.s001.docx]

**A zero-cost attention-based approach to promote cleaner streets**

Supporting Material

Rita Abdel Sater, Mathilde Mus, Valentin Wyart, and Coralie Chevallier

[**Table 1 Outcome rates, by study and colour condition** 1](#_Toc131162793)

[**Table 2 Descriptive statistics, Study 1** 2](#_Toc131162794)

[**Table 3 Descriptive statistics, Study 2.A** 2](#_Toc131162795)

[**Table 4 Descriptive statistics, Study 2.B** 3](#_Toc131162796)

[**Table 5 Descriptive statistics, Study 3** 3](#_Toc131162797)

[**Table 6 Participant exclusion rates** 4](#_Toc131162798)

[**Table 7 Mixed model Anova results for COLOUR (grey,colour) x STUDY (red, green, blue), in British sample** 4](#_Toc131162799)

[**Table 8 Mixed model Anova results for COLOUR (grey,colour) x STUDY (red, green), in British sample** 4](#_Toc131162800)

[**Table 9 Mixed model Anova results for COLOUR (grey,colour) x STUDY (red, blue), in British sample** 4](#_Toc131162801)

[**Table 10 Mixed model Anova results for COLOUR (grey,colour) x STUDY (blue, green), in British sample** 5](#_Toc131162802)

[**Table 11 Mixed model Anova results for COLOUR (grey,colour) x STUDY (british sample, French sample)** 5](#_Toc131162803)

**Table 1 Outcome rates, by study and colour condition**

|  |  | **Outcome rate** | | | | |  |
| --- | --- | --- | --- | --- | --- | --- | --- |
|  |  | **Hit** | | | **False Alarm** | |  |
|  | |  | **Grey** | **Color** | |  |  |
| **Study 1 (red)** | |  | 0.51 | 0.61 | | 0.13 | |
| **Study 2.A (green)** | |  | 0.50 | 0.62 | | 0.12 | |
| **Study 2.B (blue)** | |  | 0.52 | 0.68 | | 0.12 | |
| **Study 3 (blue – French sample)** | |  | 0.55 | 0.73 | | 0.13 | |

| **Table 2 Descriptive statistics, Study 1** | | | | |
| --- | --- | --- | --- | --- |
|  | **Overall, N = 308** |  | **Female, N = 204** | **Male, N = 104** |
|  |  |  |  |  |
| **Uses vision correction** | 60% |  | 70% | 40% |
|  |  |  |  |  |
| **Colour blind** | 1.6% |  | 0.5% | 3.8% |
| **Education** |  |  |  |  |
| Some high school or less | 1.0% |  | 1.0% | 1.0% |
| Completed high school | 15% |  | 12% | 21% |
| Some college | 19% |  | 20% | 15% |
| Completed college | 38% |  | 40% | 34% |
| Post graduate | 25% |  | 25% | 27% |
| Trade/technical school | 2.3% |  | 2.5% | 1.9% |
| **Age** Mean(SD) | 37(13) |  | 38(13) | 35(13) |
| **Discriminability**  ***d’ red*** | 1.65(0.72) |  | 1.60(0.72) | 1.75(0.70) |
| ***d’ grey*** | 1.34(0.62) |  | 1.28(0.64) | 1.46(0.57) |
|  |  |  |  |  |

| **Table 3 Descriptive statistics, Study 2.A** | |  | | |  |  |  |
| --- | --- | --- | --- | --- | --- | --- | --- |
|  | **Overall, N = 301** | |  |  | | **Female, N = 174** | **Male, N = 127** |
|  |  | |  |  | |  |  |
| **Uses vision correction** | 57% | |  |  | | 66% | 46% |
| **Colour blind** | 2.3% | |  |  | | 0.6% | 4.7% |
| **Education** |  | |  |  | |  |  |
| Some high school or less | 0.7% | |  |  | | 1.1% | 0% |
| Completed high school | 14% | |  |  | | 13% | 14% |
| Some college | 19% | |  |  | | 18% | 20% |
| Completed college | 40% | |  |  | | 40% | 39% |
| Post graduate | 26% | |  |  | | 26% | 25% |
| Trade/technical school | 1.0% | |  |  | | 0.6% | 1.6% |
|  | 0% | |  |  | | 0% | 0% |
| **Age** Mean(SD) | 36(12) | |  |  | | 36(12) | 36(12) |
| **Discriminability**  ***d’ green*** | 1.73(0.70) | |  |  | | 1.75(0.70) | 1.71(0.70) |
| ***d’ grey*** | 1.40(0.59) | |  |  | | 1.39(0.61) | 1.42(0.57) |

| **Table 4 Descriptive statistics, Study 2.B** | | | | |
| --- | --- | --- | --- | --- |
|  | **Overall, N = 308** |  | **Female, N = 201** | **Male, N = 107** |
|  |  |  |  |  |
| **Uses vision correction** | 60% |  | 65% | 51% |
| **Colour blind** | 1.6% |  | 0% | 4.7% |
| **Education level** |  |  |  |  |
| Some high school or less | 2.3% |  | 2.5% | 1.9% |
| Completed high school | 13% |  | 16% | 7.5% |
| Some college | 14% |  | 11% | 21% |
| Completed college | 42% |  | 41% | 43% |
| Post graduate | 28% |  | 30% | 23% |
| Trade/technical school | 1.3% |  | 0% | 3.7% |
| **Age** Mean(SD) | 37(12) |  | 38(12) | 35(12) |
| **Discriminability**  ***d’ blue*** | 1.86(0.71) |  | 1.80(0.69) | 1.97(0.73) |
| ***d’ grey*** | 1.38(0.63) |  | 1.34(0.63) | 1.47(0.61) |

| **Table 5 Descriptive statistics, Study 3** | | | | |
| --- | --- | --- | --- | --- |
|  | **Overall, N = 283** |  | **Female, N = 123** | **Male, N = 160** |
|  |  |  |  |  |
| **Uses vision correction** | 57% |  | 62% | 54% |
| **Colour blind** | 2.1% |  | 0.8% | 3.1% |
| **Age** Mean(SD) | 38(14) |  | 38(13) | 38(14) |
| **Discriminability**  ***d’ blue*** | 2.04(0.85) |  | 2.02(0.85) | 2.06(0.85) |
| ***d’ grey*** | 1.46(0.75) |  | 1.42(0.76) | 1.50(0.74) |
|  | | | | |

**Table 6 Participant exclusion rates**

|  |  | **Initial N** | **Failed 5 or more catch trials** | **Unrealistic Reaction time on more than 30% of trials** | **Final N** |  |
| --- | --- | --- | --- | --- | --- | --- |
| **Study 1 (red)** |  | 324 | 16 | 0 | 307 | |
| **Study 2.A (green)** |  | 312 | 11 | 0 | 301 | |
| **Study 2.B (blue)** |  | 315 | 7 | 0 | 308 | |
| **Study 3 (blue – French sample)** |  | 307 | 24 | 1 | 282 | |

**Table 7 Mixed model Anova results for COLOUR (grey,colour) x STUDY (red, green, blue), in British sample**

| ***Effect*** | ***DFn*** | ***DFd*** | ***F*** | ***p-value*** | $\boldsymbol{\eta}^{\boldsymbol{2}}$ |
| --- | --- | --- | --- | --- | --- |
| Study | 2 | 912 | 2.73 | 0.07 | 0.01 |
| Colour | 1 | 912 | 1191.40 | 0.00 | 0.57 |
| Study : Colour | 2 | 912 | 22.67 | 0.00 | 0.05 |

**Table 8 Mixed model Anova results for COLOUR (grey,colour) x STUDY (red, green), in British sample**

| ***Effect*** | ***DFn*** | ***DFd*** | ***F*** | ***p-value*** | $\boldsymbol{\eta}^{\boldsymbol{2}}$ |
| --- | --- | --- | --- | --- | --- |
| Study | 1 | 605 | 1.65 | 0.20 | 0.00 |
| Colour | 1 | 605 | 574.60 | 0.00 | 0.49 |
| Study:Colour | 1 | 605 | 0.46 | 0.50 | 0.00 |

**Table 9 Mixed model Anova results for COLOUR (grey,colour) x STUDY (red, blue), in British sample**

| ***Effect*** | ***DFn*** | ***DFd*** | ***F*** | ***p-value*** | $\boldsymbol{\eta}^{\boldsymbol{2}}$ |
| --- | --- | --- | --- | --- | --- |
| Study | 1 | 613 | 5.34 | 0.02 | 0.01 |
| Colour | 1 | 613 | 847.95 | 0.00 | 0.58 |
| Study:Colour | 1 | 613 | 35.93 | 0.00 | 0.06 |

**Table 10 Mixed model Anova results for COLOUR (grey,colour) x STUDY (blue, green), in British sample**

| ***Effect*** | ***DFn*** | ***DFd*** | ***F*** | ***p-value*** | $\boldsymbol{\eta}^{\boldsymbol{2}}$ |
| --- | --- | --- | --- | --- | --- |
| Study | 1 | 606 | 1.09 | 0.30 | 0.00 |
| Colour | 1 | 606 | 1003.81 | 0.00 | 0.62 |
| Study:Colour | 1 | 606 | 32.01 | 0.00 | 0.05 |

**Table 11 Mixed model Anova results for COLOUR (grey,colour) x STUDY (british sample, French sample)**

| ***Effect*** | ***DFn*** | ***DFd*** | ***F*** | ***p-value*** | $\boldsymbol{\eta}^{\boldsymbol{2}}$ |
| --- | --- | --- | --- | --- | --- |
| Study | 1 | 588 | 5.07 | 0.02 | 0.01 |
| Colour | 1 | 588 | 1585.63 | 0.00 | 0.73 |
| Study:Colour | 1 | 588 | 14.95 | 0.00 | 0.02 |

**Table 7 Results by gender: Linear regressions results for Studies 1, 2.A and 2.B**

|  | | | |
| --- | --- | --- | --- |
|  | | | |
|  | Dependent variable: d’ | | |
|  |  | | |
|  |  | | |
|  | Study 1  (colour: red) | Study 2.A  (colour: green) | Study 2.B  (colour: blue) |
|  | | | |
| Colour | 0.32^***^ | 0.36^***^ | 0.46^***^ |
|  | (0.07) | (0.07) | (0.07) |
|  |  |  |  |
|  |  |  |  |
| Male | 0.18^**^ | 0.02 | 0.13 |
|  | (0.08) | (0.08) | (0.08) |
|  |  |  |  |
| Colour * Male | -0.03 | -0.07 | 0.04 |
|  | (0.11) | (0.11) | (0.11) |
|  |  |  |  |
|  |  |  |  |
|  |  |  |  |
| Constant | 1.28^***^ | 1.39^***^ | 1.34^***^ |
|  | (0.05) | (0.05) | (0.05) |
|  |  |  |  |
|  | | | |
| Observations | 616 | 601 | 616 |
| R^2^ | 0.06 | 0.06 | 0.12 |
| Adjusted R^2^ | 0.06 | 0.06 | 0.12 |
| Residual Std. Error | 0.67 (df = 612) | 0.65 (df = 597) | 0.67 (df = 612) |
| F Statistic | 13.94^***^ (df = 3; 612) | 13.12^***^ (df = 3; 597) | 28.51^***^ (df = 3; 612) |
|  | | | |
|  | ^*^p^**^p^***^p<0.01 | | |
