# Supplementary material for: A zero-cost attention-based approach to promote cleaner streets: A Signal Detection Theory approach in Parisian streets
Source: PLoS One. 2023 Apr 26;18(4):e0284272. doi: 10.1371/journal.pone.0284272 (PMC10132669; doi:10.1371/journal.pone.0284272)
Supplement: Attachment — Submitted filename: Response to Reviewers.docx [file pone.0284272.s002.docx]

Dear editor,

Thank you for giving us the opportunity to submit a revised draft of our manuscript. We appreciate the time and effort that you and the reviewers have dedicated to providing your feedback on our manuscript. We have incorporated the reviewers’ suggestions and highlighted the changes in red within the manuscript.

You will find below a point-by-point response to the reviewers’ comments.

**Reviewer #1:  While the topic of the study was exciting, the results do not warrant publication in their current form. Listed below are my key concerns, and unfortunately, I do not believe that these issues can be addressed without additional data.**

**1. The critical missing piece is data showing the connection between bin density and litter. The authors make a fundamental assumption that higher bin density will result in lower littering rates, but this is not substantiated. In fact, from the social psychological research on normative influence, there is reason to predict that increased bin density could spark MORE litter in environments that have a high degree of existing litter (see research by Cialdini on the focus theory of normative conduct).**

The focus theory of normative conduct predicts that a higher number of bins or more visible trash cans, coupled with high levels of litter, might induce increased littering. However, empirical studies suggest that making trash containers more attractive or noticeable can work as an effective alternative to adding bins (Arnold, 2015; Geller et al., 1980; O’Neill, Blanck & Joyner, 1980). More visible bins are also used more, regardless of their availability, which suggests that an increase in saliency can be an effective policy to decrease littering down the line. Our goal was to provide an objective way of measuring which colours are more likely to increase salience.

We altered the sentence in the introduction to include Cialdini’s reference 5 (line 51):

*“While some theories suggest that an increase in the number of bins might increase litter in the presence of litter in the environment (11), most empirical studies that have focused on understanding and preventing the widespread prevalence of littering as early as in the 1970s have confirmed that sites with more receptacles tend to have lower littering rates (12–15).”*

**2. More clarity is needed about the theoretical foundations of this research. The authors briefly mention the Signal Detection Theory, and later 'Nudge Theory', yet the results are not integrated into these two foundations. Specifically, Study 3 revealed the strongest increases in perceptions of receptacles as a result of switching to blue bags, compared to green and red bags. However, the authors do not address that this result is contradictory to the signal detection theory's claims for increased attention to the color red.**

To the best of our knowledge, signal detection theory does not claim that the colour red necessarily increases attention. Exogenous attention is referred to as bottom-up salience and is typically triggered by contrast differences between objects and their surroundings, e.g., if the colour of an object is particularly salient compared to the background against which it stands. Therefore, a blue object among various red objects will stand out more than a red object.

We clarified this in the introduction (line 94):

*“Exogenous attention is also referred to as bottom-up salience and typically emerges from contrast differences between objects and their surroundings; for example, if the colour of an object is particularly salient compared to the background against which it stands (e.g., a blue object against a red background will be salient).”*

**In addition, while Nudge Theory is cited, there is a body of research in social psychology around norms that is highlight relevant and should be integrated into the paper.**

Nudges offer ways to alter the surroundings of individuals and directing them towards the desired outcome using cognitive levers such as priming (lemon scented trains to decrease litter (DeLange et al., 2012) or social norms (displaying eye images to decrease litter (Bateson et al., 2011)). We agree that social psychology also provides a relevant body of research which is now cited more extensively. However, we would like to emphasize that our method focuses on a measure of exogenous attention, which is likely less susceptible to social norm effects.

However, we acknowledge the Reviewer’s comment and add the following sentence to further clarify things (line 100):

*“Interventions in litter control, such as ex-ante campaigns reminding individuals of littering costs or highlighting the social norm or high fines that punish uncivil behaviour ex-post, might increase endogenous attention and trigger top-down visual search to find the closest bin (23,24). Interventions aiming at increasing the salience of bins relative to the surroundings will, on the other hand, trigger automatic attentional capture to the bin. Such an intervention could take the form of a simple change of trash bag colour from grey to more salient colours, leading to an increase in the perceived number of bins.”*

**3. One of the implications of this research is support for supplementing the cost associated with increased trash bins compared with increased salience of existing bins. However, the authors mention that the city previously utilized green bags but switched to grey bags to reduce the appearance of trash receptacles in crowded public spaces. Thus, this study's findings are in opposition to the intended application by the City of Paris.**

In the touristic city of Paris, there is a trade-off between the aesthetics of the bins and litter control, and the color of bins should be changed most efficiently to maximize the visibility for the aesthetic cost. There is an ongoing debate between officials who vouch to increase the number of bins or to make them more visible and those who vouch for making bins more invisible for aesthetic reasons. Even though the city of Paris has previously switched the colour of bin bags to grey, there was no evaluation on whether this had produced more or less litter, and as the deliberation is ongoing, the city was interested in having more evidence to figure out whether this had had any effect on the visibility of bins.

**4. The authors use the phrase 'perceived trash density' to refer to the salience of trash bins, but this term mischaracterizes the physical norm. Trash density could be misinterpreted to refer to the amount of litter or trash in the physical environment. Instead, the authors should refer to this as perceived receptacle density.**

We thank the reviewer for pointing out this error. The occurrence of *“perceived trash density”* line 110 was changed to *“perceived bin density”* to match the rest of the sentences in the paper.

**Reviewer #2:**

**This article reports the findings from three studies that assessed the impact of bin bag color on bin visibility in an online experimental setting. Using a Signal Detection Theory approach and photographs featuring bins on urban Parisian streets as stimuli, the authors tested whether changing the bin bag color from grey to red, green or blue increased bin detection rates. Studies 1 and 2 involved British participants to infer a common nationality of tourists to Paris, whereas Study 3 featured a more local Parisian sample. I found this article an interesting read, and I believe it addresses an important gap in literature in the area of environmental psychology and policy. The authors have provided a convincing example of a series of online studies that lends itself to cost-effective implications in real-life contexts. However, I have identified four main areas that require revision before the article can fulfill the criteria of publication in this journal. These revisions do not require extensive adjustments to the manuscript, and thus I have recommended minor revisions.**

**CRITICAL APPRAISAL**

**The authors haven’t discussed limitations in regard to the methodology that they have used. The studies could be seen as very leading and lacking ecological validity based on the design (i.e. telling people to look for bins and stating if they have seen them vs. not). They do note analytical techniques to try and reduce biases, but this also needs to be acknowledged in the discussion. At the moment, it implies that the findings show detectability, but that cannot be assumed to translate to everyday experiences of the Parisian streets. It would have been nice to have a field study to test it in a real life context or a laboratory study that has a less obvious leading focus, but at least the authors should acknowledge the barriers of conducting an experimental (online) study and its implications. In summary, more discussion and critical appraisal of the aforementioned points would make the article a more sound and convincing piece of scientific research.**

We thank the reviewer for this comment. We would like to stress that the aim of the study was to tap bottom-up attention, or how much is a bin seen when the passerby is precisely *not* looking for one. A very short display time helps us tap this process as the person does not have time to direct their eyes more than 2 or 3 times. In the discussion, we acknowledge that we cannot infer much about real-life conditions where people are actively looking for a bin and leverage top down attention. Additional field experiments are needed to test the combined effects of bottom-up and top-down attention.

This was clarified in the discussion (line 425):

*"Our results also provide grounds for future work investigating whether an increased visibility of bins has a subsequent impact on littering behaviour but a number of limitations should be acknowledged. First, our set of experimental online studies may lack ecological validity and looking for a bin in real settings may rely on different mechanisms. Specifically, the short display time in our experiment mimics the impact of increased visibility when people are not actively looking for a bin, which differs from what happens in real-life. Moreover, our studies do not allow us to state whether changing bag colour would have an effect on real-life littering. However, prior research provides suggestive evidence that making bins more attractive or noticeable affects behavior (17–19).”*

**LACK OF REFERENCES**

**The Introduction makes a strong case for the rationale of the study, and the authors have discussed the impacts of litter in the environment convincingly. Similarly, discussion on implications is thorough and plausible. In my view, some of the key claims, especially those in the Introduction (e.g. information about Paris’ waste management system and budget, endogenous and exogenous attention) and sections of the methods (e.g. when justifying bin bags colours) would need to be supported with references. I would recommend that the authors provide more justification to their claims by providing more references to existing literature throughout.**

The following references have been added in support of the claims made by the authors.

*“La droite donne un an à Hidalgo pour améliorer la propreté de Paris [Internet]. Les Echos. 2018 [cité 1 sept 2021]. Disponible sur:* [*https://www.lesechos.fr/2018/02/la-droite-donne-un-an-a-hidalgo-pour-ameliorer-la-proprete-de-paris-983415*](https://www.lesechos.fr/2018/02/la-droite-donne-un-an-a-hidalgo-pour-ameliorer-la-proprete-de-paris-983415)*”*

*« Berger A, Henik A, Rafal R. Competition Between Endogenous and Exogenous Orienting of Visual Attention. Journal of Experimental Psychology: General. 2005;134(2):207‑21. »*

We cannot provide more references about the choice of colours since it came from exchanges during meetings with the local authorities with whom we worked on this project.

**Finally, it is surprising that nudge theory was only mentioned in the Discussion, and not in the Introduction, so an earlier reference is needed.**

Indeed, it would be useful to mention nudge in the introduction to contextualise the study. The following sentence was added in the introduction (line 74):

“In line with research on nudges, such simple changes to the physical surroundings can be effective in significantly changing behaviour (20)”

**OMISSIONS/INCONSISTENCIES IN THE RESULTS SECTION AND DATA ANALYSIS**

**In general, the Method and Results sections read well and are concise, and the provided photographs that help to illustrate the experimental design. The authors have also explained the application of the methodology in the current studies very well. However, the authors’ statement of statistical power (Method section) is incoherent and superficial, as it is unclear what “a minimum effect of 10%” means. PLOS ONE guidelines state that “If a sample size calculation was performed, specify the inputs for power, effect size and alpha”.**

We acknowledge that the power calculation reporting was opaque. The following paragraph was added to clarify (line 203):

“...*which is a large enough sample to detect a small effect size of dz=0.15 (α=5%, 1-β=80%) following a power analysis with G*Power (version 3.1.9.3) for a two-tailed t-test for dependent means, and using outcome variances computed in the pilot study over 100 participants.”*

**Regarding how the authors have reported their findings, I think it is important that the statistical tests are mentioned each time the results are reported (as currently ‘paired t-tests’ is only mentioned in Results for study 1)**.

We thank the reviewer for drawing our attention to this oversight, which is now corrected in the Result sections of Studies 2.A, 2.B and 4.

**“and past tense should be used when reporting findings.”**

The present tense was changed to the past tense in all result sections.

**“As PLOS ONE asks for effect sizes, these should be provided, or a justification as to why they haven’t been provided in this instance should be given. As this paper seems to be directed to decision makers, it would also be useful to qualitatively state the effect size (e.g. d = xx, a medium effect) to help emphasize the strength further.”**

We added all effect sizes to the results sections.

“...(cohen’s d=0.88, a large effect)” (line 252)

“...(cohen’s d=0.45, a medium effect)” (line 255)

“...(cohen’s d=1, large effect size)” (line 299)

“This is a very large effect size with Cohen’s d for repeated measures equaling 1.55.” (line 307)

“(cohen’s d for repeated measured=1.5, large effect size)” (line 375)

**The largest omission within the results section is the detailed given for the ANOVAs. The results from the mixed model ANOVAs are not reported in enough detail to follow the analysis, know what was done and for the reader to conclude which color bags produced the ‘larger’ changes from grey. Although the authors provide the d’ values earlier in the results section, it would still be useful to state here the directions of the interaction effects and report post-hoc analyses if relevant. Figure 4 helps with this interpretation, but clearer (and higher resolution) interaction plots could be provided to help illustrate results from the mixed model ANOVA.**

We acknowledge the results concerning the mixed ANOVA were unclear. To help illustrate the results, we made adjustments to the results section and replaced the figure with the following interaction graph, as suggested by the reviewer.

**Fig 4. Interaction graph showing the change in detectability when a bin is coloured, in studies 1 and 2.** Mean and 95% confidence interval.

**The authors should also note that the Interpretation of the findings for reaction times on page 16 seem incorrect, stating reaction time was shorter for the colored condition, but then Study 2B the colored condition was slower (1056 seconds vs. 1006 seconds).**

We thank the reviewer for pointing out this mistake. We had inverted the previously erroneous reaction times and have now corrected this in the manuscript.

**Moreover, while all other studies report what the equivalent of the percentage difference would be in terms of number of new bins installed in Paris, this information is not provided for the blue bag condition (Study 2B).**

We thank the reviewer for pointing out this unintentional omission. We have added the following (line 311):

*This translates into an increase of 46% of trash can visibility, similar to the addition of 13,600 new bins.”*

**Another key point is that the authors have referred to potentially new findings in the Discussion (asking participants which color they would pick). Is this already published, thus needing a citation, or is this unpublished work? This information either needs to be citeable or included as a study in the paper, as no new findings should be introduced in the Discussion.**

We added a section in the paper to report these findings as a separate study.

*“****Study 3: Which colour would you have picked?***

*The superiority of the colour blue to the colour red in the bin detection tasks did not follow our initial intuition, that the colour red would be most visible. We thus ask a random sample of participants which colour they would choose if they were policy makers. This could be an important point since policy makers often base important choices on intuition, which might be highly inefficient.*

***Participants and trial***

*160 British nationals were recruited on the platform Prolific Academic and received a compensation of 62 euros per hour. Participants were only asked one question: “Imagine you are in charge of making sure that bins in your city are highly visible. Which colour would you pick for the bins? Green, red or blue?''. The choices appeared in a random order to participants in order to avoid any biases in answers.*

***Results and Discussion***

*The most common answer was red (50%), followed by blue (32%) and green (18%). This was consistent with our intuition and some studies on visual attention that red is believed to be the most salient (1). Assuming that policy makers’ intuition is comparable, this might lead Parisian policy makers to choose a sub-optimal solution when choosing the colour that best increases the saliency of a bin in the city.”*

Additionally, we changed the paragraph in the discussion, to:

*“Indeed, if a policy maker wants to increase the perceived density of bins, it would be inefficient to go straight to a costly field trial comparing various bin colours. And as our additional data show, it would be even more inefficient to simply rely on intuitions. When we asked a sample of different participants what colour they believe would be the most efficient at increasing bin visibility, the majority answered “red” while our studies show that blue is in fact the colour that best improves detectability. Determining which colour is most likely to be salient in a particular city in an experimental setting first can therefore be a useful evidence-based first step before elaborating a field strategy.”*

**WRITTEN EXPRESSION AND FORMATTING**

**The paper is mostly written in clear and consistent way. However, the authors should note that “zero-cost” is a strong statement for this paper. As the purpose of the paper wasn’t to run a cost-benefit analysis, it looks like this is based on an assumption (for example, might colored bags be charged differently, need different suppliers, harder to source?).**

We acknowledge that the term “zero-cost” might not be generalizable to all contexts. However, in the context of this study, our exchanges with the local authorities confirmed that the red, green and blue bags were provided by the same suppliers and cost the same as grey bags, so if the authorities changed their bags from grey to red, the cost would truly be zero.

*“Footnote 1. In the context of this study, our exchanges with the Parisian local authorities confirmed that the red, green and blue bags were provided by the same suppliers and cost the same as grey bags, so if the authorities changed their bags from grey to red, the cost would be zero. This, however, does not necessarily hold in all contexts where the production of a new coloured bag might incur a relatively low, but non-zero cost.”*

**It is also important to use neutral terms (“manpower” could be changed to ‘resources’ in the Introduction) and avoid idiomatic expressions (“down-the-road” could be changed to ‘subsequent’ in the Discussion).**

We thank the reviewer for these suggestions. The two substitutions have been applied as suggested.

**Similarly, “promising” (p. 9) needs to be elaborated on for clarity as in its current format it appears contradictory.**

We agree with the reviewer that the word might create confusion. It has been substituted with “relevant”.

**In regard to formatting, use of line numbers and double spacing throughout would have made the article easier to read and review.**

**Similarly, it is important to be consistent with formatting of paragraph transitions.**

The formatting has been adjusted accordingly and line numbers + double spacing have been applied.

**Finally, the paper should be checked for typing errors and clarity – Some typos, formatting errors and other points that need elaboration are listed below:
\the and /word” (\ is sometimes used in place of “)**.**SD should be used instead of +/- (when reporting age of participants)**.**p <:001 should be p < 0.001 (PLOS ONE guidelines)**.**be consistent with reporting the statistics – 2 decimal points**.**close bracket missing on page 16**. **Some numbers don’t add up (p. 12 (104-204 = 309) might mean one participant did not report their gender, but needs to be stated.)**. **As the studies were not ran under highly controlled laboratory conditions, they should not be referred to as laboratory studies (maybe experimental online studies)**

All of the typos have been corrected and all suggestions were taken into account and altered in the text.

**Paragraph 5/6 in the Introduction: Should be reworded to better reflect that bins that blend with the environment are NOT a good idea.**

Indeed, the paragraph should be rephrased in order to better reflect the trade-off. The last two sentences were changed to (line 78):

*“This solution appears particularly relevant in cities, where policy makers strive to keep streets charming and deliberately favour designs that make bins blend with the surrounding urban environment. In Paris, for instance, public trash bins are made of a light grey metal and fitted with discreet grey bags, which means that they are essentially designed to be invisible (Fig 1).”*

**Were people who reported being color blind excluded from the study? There’s reference this information was collected, but did not state how the authors used it.**

Color blind participants were not excluded from the analysis, since we believe this improves the generalizability of the results to real-life situations. The results were used to make sure that the rate of colour-blind participants was not higher than the one found in the population.

We clarified this point in the paper in all “Participants” sections:

*“The share of color blind participants was similar to that observed in the United Kingdom.”*

**Abstract: It is not clear to a reader who hasn’t read the full text whether the “replication studies” mentioned at the end are included in or separate from the “three pre-registered studies” mentioned at the beginning.**

The abstract was changed to clarify this point:

*“In an effort to inform interventions targeting littering behaviour, we estimate how much a change in trash-bag colour increases trash can visibility in Paris. To that end, we applied standard Signal Detection techniques to test how much changing trash-bag colour from grey to red affects subjects' detection rates. In three pre-registered studies (total N = 922), we found that changing trash bag colour considerably increases the perceived number of bins: a change from grey to red translates into a 28% increase in the perceived number of bins (Study 1). When considering changes in bottom up attention, this means that a zero-cost change of trash-bag colour from grey to red could be equivalent to installing up to 8,400 additional bins in the city of Paris, in terms of perceived density. Two replication studies investigating other colour changes showed that changing the colour from grey to blue further increases visibility, with blue exhibiting the highest increase in visibility in a sample living in the Paris area.”*

**Reviewer #3: The manuscript reports an experiment based on internet data collection on a visual detection task of trash bins within a complex visual scene. The experimental design is a within-subjects in which the colour of the visual target is manipulated. The results show that when the target is coloured the sensitivity of the subject is higher than when the target is grey. The result is replicated with different colours, and it turns out that blue is the one that favours a higher detectability of the target. Furthermore, a final study shows that the effect is present regardless of whether participants are previously familiar with the target or with the complex visual scene.**

**The idea that colour shade can influence the detection (and identification) of visual stimuli is not particularly new, but the scope of this study is. I found the manuscript interesting and well written. The methodology is sound. In fact, the visual detection paradigm is applied to a realistic context, the images are cityscapes of Paris, and the target are the city's trash bins. I agree with the authors on the relevance of the study which demonstrates how changing the colour of the bin bags from the current grey to blue can increase bin detection and potentially improve urban decency.**

**However, I feel that some of the claims (particularly in the discussion) are speculative. Although the dependent variable is the detection accuracy for individual bins in cityscape images, the authors refer several times in the text to the perceived density of bins. Since the latter is never measured directly or indirectly, I think the authors should revisit the claim that changing the colour of the bag produces an increase in the perceived density of the bins. If the authors assume a linear relationship between detection accuracy and the perceived density of the bins in the visual scene, they should at least substantiate this relationship with other data or with support from previous literature, since they only have one bin per image in this experiment. Perceived density may in fact depend not only on detectability, but also on salience and other contextual factors. The same applies to the conversion between the increase in detection accuracy and the absolute number of bins in the city and consequently also to the equivalent economic savings.**

**In fact, the data analysed refer to two-dimensional images with exposure times of 700 milliseconds. as the authors were interested in measuring the bottom-up effect related to colour. What would happen without such high time pressure? In a real-life situation there is usually sufficient time for serial scanning of scene elements and this could greatly reduce the difference in detection rate between coloured and grey bins. Therefore, quantifying economically the savings in terms of number of bins based on the data of this experiment which is limited to a very specific condition could be misleading. It is not known how much the detection rate would increase under real conditions, nor the perceived density of bins (in the virtual or real environment). Although reporting how many euros would be saved is suggestive, the exact amount remains speculative. I would recommend the article for publication only after the authors have more cautiously revised the sentences that are not directly supported by the experimental data.**

We agree with the author that these claims are speculative and they are only for making concrete comparisons to policy makers, but are based on assumptions. The tone of the discussion has thus been changed to imply this, and the approximated economic gains have been removed throughout the study (line 405):

*“The results of this study suggest that a mere change in the colour of bins can result in a cost-effective intervention that would increase the visibility of bins. This implies that some previously invisible bins would become visible as a result of the intervention, thereby increasing the perceived density of bins in an urban setting. Assuming a linear relationship, and for illustration, we speculate that a change of bag colour from grey to red, green or blue in the city of Paris could be equivalent to installing up to 8,370, 9,537 or 13,600 additional bins, respectively.”*

Additionally, in the abstract, the phrase *“considerably increases the perceived density of bins”* was changed to *“considerably increases the perception of bins”*

**Have you checked that the assumptions for ANOVAs were met? You should report this in a sentence in the analysis section.**

We checked that the ANOVA assumptions of normality, independence of samples, and sphericity were met. However, we did not have homogeneity of variances at all treatment level (Levene’s test, p < .05). We therefore performed a robust ANOVA using the R package WRS2. The results section was fixed accordingly (line 319):

*“The ANOVA assumptions of normality, independence of samples and sphericity were met but variances were heterogeneous. We therefore ran a mixed-model robust ANOVA using the R package WRS2, with 3-level factor STUDY (green, red and blue) and 2-level factor COLOUR (grey, colour).”*

**You should also report if any correction has been applied and you should also better specify the t.test (is it always equal variance two-tailed t-test?). When doing multiple test on the same dataset you should perform a correction for multiple comparisons (for example when testing green vs red vs blue in pairs).**

All instances of t.test were substituted for "*paired equal variance two-tailed t-test for repeated measures*” to better specify the t-test conducted.

**Sometimes it is not clear which are the factors and the levels of the factor in the ANOVAs: In the ANOVA for COLOUR x STUDY, how many COLOUR factor levels are there? One would expect a factor with 3 levels (red, green, and blue) and thereafter a series of pair-wise post-hoc with correction for multiple comparisons, but you seem to have done 3 separate ANOVAs, is there a particular reason?**

The reason why 3 separate ANOVAs with 2 factors were conducted after the ANOVA with 3 factors was to be able to capture a change in the "effect size" or the difference between grey and color between two factors. A pairwise post hoc test would allow a comparison of the three colors at each condition (color and grey) but not of the change from grey to color at each factor level.

We hope to have clarified this in the paper (line 317):

*" We performed a mixed-model ANOVA to compare the results obtained in Studies 1, 2.A and 2.B, using the change from grey to colour as a within subject factor and study number as the between subjects factor. The ANOVA assumptions of normality, independence of samples and sphericity were met but variances were heterogeneous. We therefore ran a mixed-model robust ANOVA using the R package WRS2, with 3-factor levels STUDY (green, red and blue) and 2-factor levels COLOUR (grey, colour). We found a significant interaction F(912)=22.672 p=0.002. We then did three separate mixed ANOVAs to compare the effect size of COLOUR, this time with a 2-factor levels STUDY. An interaction graph (Fig 4) showed the change from grey to colour across all three studies. We found significant COLOUR x STUDY interactions when comparing red (Study 1) to blue (Study 2.B) F (1, 615) =35.92 p<0.001 and when comparing green (Study 2.A) to blue (Study 2.B) F (1, 610) =32.01 p<0.001; but no interaction when comparing red (Study1) to green (Study 2.A), F (1, 611) =0.46 p = 0.45. "*

**In experiment 3 which are the factors (and the levels for each factor) for the mixed ANOVA?**

We thank the reviewer for his observation. The following was added to explain further (line 391):

"*A mixed model ANOVA testing 2-level COLOUR factor (grey vs colour) x 3-level STUDY factor (study 2.B vs study 4).*"

Minor

**I think there are some inconsistencies in the notation (e.g. sometimes d'0, .d0, d0, etc.)**

All annotations were changed to $d^{'}$.

**I think that the dot and not the colon should go before the decimal part of the p value.**

We thank the reviewer for pointing this out. The colon was changed into a dot.

**When the SDT indices are described, there are slashes instead of quotation marks.**

We corrected this unintentional typo.

**The d'0 represents the sensitivity of the participant to the stimulus. Indeed, for the same signal-to-noise ratio d'0 can vary from participant to participant. Although d' tends to increase as the signal-to-noise ratio increases (the signal and noise probability curves get further apart) I would not expressly say that d' represents the signal-to-noise ratio, but rather the sensitivity of the measuring instrument or the person making the detection.**

We agree that this needed to be reworded (line 224):

*“A discriminability of 0 means that the signal is not distinguished from noise, while higher d0 values represent a higher sensitivity (i.e. a situation where it is easier for people to discriminate the bin in the scene)”*.

**It is true that “participants did not have enough time to fully scan the scene in a top-down fashion” but at the same time the task was based on a voluntary active search involving also endogenous attention. From the introduction it seems that the task focuses on endogenous attention, but endogenous attention takes about 300 ms to be deployed and can be sustained at will whereas exogenous attention takes only about 100 ms to be deployed and it is transient (for reviews see Carrasco 2015 or Giordano 2009). Given that average reaction times are above one second this would suggest a deliberate serial scanning of the visual scene, but the fact that there is an increase in performance and also a reduction in reaction times in the 'colour' condition suggests a greater involvement of exogenous attention.**

**It remains to be seen whether prolonging the exposure time further will cause the advantage to lapse (how is the performance in catch trials?). In real-life situations we rarely have less than a second to identify the bin. I think the part about attention and reaction time is lacking in the discussion.**

We agree with the reviewer that our data suggest a greater involvement of exogenous attention, which is consistent with our interpretation and task design. Our goal was to focus on automatic and rapid attentional processes so we made sure that the stimulus presentation time only enabled 2 to 3 fixations (700 ms). Given the complexity of the visual scene, participants did not have enough time to fully scan the scene. Reaction times were indeed above one second on average, but reaction times capture several processes beyond attention, including the selection of motor goals and motor planning. We clarified this in the Results and Discussion section of Study 1 (line 258):

*“However, it is worth pointing out that the reaction times capture several processes beyond attention, including the selection of motor goals and motor planning, which explains why they are on average above one second even though attentional capture happens much faster.”*

We also would like to stress that the aim of the study was to tap bottom-up attention, or how much is a bin seen when the passerby is precisely *not* looking for one. In the discussion, we acknowledge that we cannot infer much about real-life conditions where people are actively looking for a bin and leverage top down attention. Additional field experiments are needed to test the combined effects of bottom-up and top-down attention.

This was clarified in the discussion:

*"Our results also provide grounds for future work investigating whether an increased visibility of bins has a subsequent impact on littering behaviour but a number of limitations should be acknowledged. First, our set of experimental online studies may lack ecological validity and looking for a bin in real settings may rely on different mechanisms. Specifically, the short display time in our experiment mimics the impact of increased visibility when people are not actively looking for a bin, which differs from what happens in real-life. Moreover, our studies do not allow us to state whether changing bag colour would have an effect on real-life littering. However, prior research provides suggestive evidence that making bins more attractive or noticeable affects behavior (17–19).”*

**The euro symbol is not always written correctly and sometimes the thousand separator is missing in numbers**

We thank the reviewer for pointing that out. With the removal of the figures related to financial gains, this has been adjusted in the manuscript.
