# Supplementary material for: A zero-cost attention-based approach to promote cleaner streets: A Signal Detection Theory approach in Parisian streets
Source: PLoS One. 2023 Apr 26;18(4):e0284272. doi: 10.1371/journal.pone.0284272 (PMC10132669; doi:10.1371/journal.pone.0284272)
Supplement: Attachment — Submitted filename: Response to Reviewers.docx [file pone.0284272.s003.docx]

Dear editor,

Thank you for giving us the opportunity to submit a revised draft of our manuscript. We appreciate the time and effort that you and the reviewers have dedicated to providing your feedback on our manuscript. We have incorporated the reviewers’ suggestions and highlighted the changes in red within the manuscript.

You will find below a point-by-point response to the reviewers’ comments.

**Reviewer #2: The authors have put considerable effort into revising their manuscript “A zero-cost attention-based approach to promote cleaner streets”, and as a result, the paper has improved. As mentioned in my first review, this paper provides a novel contribution to literature, and the rationale and implications of the conducted research are now discussed in a more convincing manner in the revised version. More revisions are needed (specifically relating to the stats and some areas needing further elaboration), therefore I have recommended minor revisions.**

**There are some inconsistencies in the Results reporting which I would like to draw the authors’ attention to. First, the authors need to clarify how they obtained the reported Cohen’s d effect sizes. The reported effect sizes do not match my own calculation of Cohen’s d when using the provided means and SD and exceed the feasible range (and thus the reportings of large effect sizes are also not in line with the data).**

We thank the reviewer for this remark that needs clarification.

The formula used for the Cohen’s d effect size is the one for paired samples, as the two conditions are repeated measures/within-subject. The effect size estimate Cohen's dz can also be calculated directly from the paired sample t-value and the number of participants using the formula provided by Rosenthal (1991):

$d_{z}=\frac{M_{diff}}{\sqrt{\frac{\Sigma(X_{diff}-M_{diff})2}{N-1}}}$

Or equivalently,

$d_{z}=\frac{t}{\sqrt{n}}$

Given the direct relationship between the t-value of a paired-samples t-test and Cohen's dz.

This was double checked using the command CohensD in the Package ‘lsr” in R software (<https://cran.r-project.org/web/packages/lsr/lsr.pdf>).

To clarify this, we add the previous equation to the manuscript, as well as the calculated standard deviation of the difference for each result:

*“Footnote: The formula used for the Cohen’s d effect size is based on calculation by Rosenthal (1991);* $d_{z}=\frac{t}{\sqrt{n}}$ *, given the direct relationship between the t-value of a paired-samples t-test and Cohen's dz*.*”*

**Second, more information is still needed for the ANOVA results. Specifically, the reporting of factor levels doesn’t seem consistent: row 326 ->: why is STUDY now a 2-level factor?**

The reason why 3 separate ANOVAs with 2 factors were conducted after the ANOVA with 3 factors was to capture a change in the "effect size" or the difference between grey and colour between two factors (ie. between (grey vs red) vs. (grey vs blue) vs (grey vs green). A pairwise post hoc test would allow us to compare the three colours for each condition (colour and grey) but not the change from grey to colour for each factor level.

We hope that we clarified this rationale by adding the following sentence:

“We then did three separate mixed ANOVAs to compare the effect size of COLOUR, this time with a 2-level factor STUDY, in order to measure whether there is significant interaction between each pair of studies”.

**row 393 ->: “3-level STUDY factor (study 394 2.B vs study 4)”).**

We thank the reviewer for pointing this out. It has been corrected to:

*“2-level STUDY factor (Study 2.B vs Study 3).”*

**All effects need to be fully reported: the main effect of STUDY and COLOUR as well as the currently reported interaction.**

We added the following tables to the Supplementary materials document:

**Table 2 Mixed model Anova results for COLOUR (grey,colour) x STUDY (red, green, blue), in British sample**

| ***Effect*** | ***DFn*** | ***DFd*** | ***F*** | ***p-value*** | $\eta^{2}$ |
| --- | --- | --- | --- | --- | --- |
| Study | 2 | 912 | 2.73 | 0.07 | 0.01 |
| Colour | 1 | 912 | 1191.40 | 0.00 | 0.57 |
| Study : Colour | 2 | 912 | 22.67 | 0.00 | 0.05 |

**Table 3 Mixed model Anova results for COLOUR (grey,colour) x STUDY (red, green), in British sample**

| ***Effect*** | ***DFn*** | ***DFd*** | ***F*** | ***p-value*** | $\eta^{2}$ |
| --- | --- | --- | --- | --- | --- |
| Study | 1 | 605 | 1.65 | 0.20 | 0.00 |
| Colour | 1 | 605 | 574.60 | 0.00 | 0.49 |
| Study:Colour | 1 | 605 | 0.46 | 0.50 | 0.00 |

**Table 4 Mixed model Anova results for COLOUR (grey,colour) x STUDY (red, green), in British sample**

| ***Effect*** | ***DFn*** | ***DFd*** | ***F*** | ***p-value*** | $\eta^{2}$ |
| --- | --- | --- | --- | --- | --- |
| Study | 1 | 613 | 5.34 | 0.02 | 0.01 |
| Colour | 1 | 613 | 847.95 | 0.00 | 0.58 |
| Study:Colour | 1 | 613 | 35.93 | 0.00 | 0.06 |

**Table 5 Mixed model Anova results for COLOUR (grey,colour) x STUDY (blue, green), in British sample**

| ***Effect*** | ***DFn*** | ***DFd*** | ***F*** | ***p-value*** | $\eta^{2}$ |
| --- | --- | --- | --- | --- | --- |
| Study | 1 | 606 | 1.09 | 0.30 | 0.00 |
| Colour | 1 | 606 | 1003.81 | 0.00 | 0.62 |
| Study:Colour | 1 | 606 | 32.01 | 0.00 | 0.05 |

**Table 6 Mixed model Anova results for COLOUR (grey,colour) x STUDY (blue, green), in British sample**

| ***Effect*** | ***DFn*** | ***DFd*** | ***F*** | ***p-value*** | $\eta^{2}$ |
| --- | --- | --- | --- | --- | --- |
| Study | 1 | 588 | 5.07 | 0.02 | 0.01 |
| Colour | 1 | 588 | 1585.63 | 0.00 | 0.73 |
| Study:Colour | 1 | 588 | 14.95 | 0.00 | 0.02 |

**The justification of the follow-up analysis needs to also be given: by running separate mixed ANOVAs, familywise error is not being controlled for. Either consider running post-hoc analysis that is appropriate for these data, or provide a rationale for the chosen analysis.**

Considering that we have 3 separate mixed ANOVAS, after Holm-Bonferroni corrections, adjusted p-values are:

p-values = 3.49e-09 (Study 1 vs Study 2.B) , 2.37e-08 (Study 2.A vs Study 2.B) , 0.498 (Study 1 vs Study 2.A)

Holm-Bonferroni adjusted p-values = 1.047e-08, 3.555e-08, 0.498

As a result, the following sentence was added to the manuscript.

*“We verify that the results were still significant after Holm-Bonferroni corrections for familywise error”.*

**Finally, the statistics need to be fully reported (e.g. following APA guidelines, such as providing the effect size (partial eta squared)).**

We added partial eta squared results and properly reported statistics following the APA guidelines throughout the results sections.

**In terms of the reviewed literature and theory, the authors have now mentioned nudges in the Introduction, which is a necessary addition. However, please provide more elaboration around nudging and related literature. The paragraph starting on line 72 sounds crucial for this paper, but more elaboration is needed (a more appropriate level of depth can be found in the discussion, but this also needs to come earlier to set the scene of the paper).**

In order to elaborate around the concept of nudging, we have reordered the paragraph and added two examples from the literature on changing the physical environment:

*“In line with research on nudges, such simple changes to the physical surroundings can be effective in significantly changing behaviour (17). For example, introducing the scent of lemon in train carriages led to a decrease in litter, suggesting commuters were primed with clean-environment cues (18). Similarly, a number of studies showed that positioning healthy foods and snacks at eye level more often draws attention to that option and ultimately increases healthy food consumption (19). Current evidence shows that a more visible bin is more used, regardless of its positioning, which suggests that increasing saliency can be an effective policy to decrease littering (20–22). This solution appears particularly relevant in cities, where policy makers strive to keep streets charming and deliberately favour designs that make bins blend with the surrounding urban environment.”*

**Some areas of the text also need more clarification. For example, how many studies are being reported and the role of each one is not clear throughout the paper (e.g. in the abstract and end of the introduction). From my interpretation, there are five studies: three that tests detectability of bins using different colored bags on a British (tourist) population, another to test on a different population (residents) and one perception study. This needs to be made clearer throughout and would recommend avoiding the term “replicability studies” as I believe the studies all have their own unique contribution to promote and thus is more than just a “replicability study”.**

We agree with the reviewer that the phrasing was confusing.

We have changed the abstract to:

*“In an effort to inform interventions targeting littering behaviour, we estimate how much a change in trash-bag colour increases trash can visibility in Paris. To that end, we applied standard Signal Detection techniques to test how much changing trash-bag colour affects subjects' detection rates. In three pre-registered studies, we found that changing trash bag colour from grey to either red, green or blue considerably increases the perception of bins in British (tourist) and Parisian (resident) samples. We found that changing the bag colour from grey to blue increased visibility the most.”*

We also changed the last part of the introduction to:

*“Using a signal detection task applied to modified photographs of Parisian streets, we ran three studies to measure the impact of simple changes in trash-bag colour on bin detection. Studies 1 and 2 examined the change in trash-bag colour from grey to either red, green or blue on a British sample and Study 3 examined the change from grey to blue, this time on a sample of Paris residents.”*

Moreover, all instances of replicability or replication were substituted with *“follow-up study”*.

**Finally, there are a few minor omissions and typing errors that need to be resolved:**

**- Avoid non-scientific writing (e.g. lines 269-27, 273-274, 339) – decisions should be based on evidence, not intuition or what is thought to be “interesting”**

We thank the reviewer for pointing out this non-scientific reporting issue. We corrected the paragraph as follows:

*“We apply the same tools and methodology to other colors most frequently considered in the visual saliency literature, blue and green (34,35). These two colors also bear practical importance for the current study, since they have been considered by the Parisian municipality as potential alternative trash bag colors. In fact, green bags were used by the city from 2013 up until 2019 and were then replaced by grey bags for aesthetic reasons.”*

*34. Mullen KT, Kingdom F a. A. Differential distributions of red–green and blue–yellow cone opponency across the visual field. Visual Neuroscience. janv 2002;19(1):109‑18.*

*35.	Kamkar S, Moghaddam HA, Lashgari R. Early Visual Processing of Feature Saliency Tasks: A Review of Psychophysical Experiments. Frontiers in Systems Neuroscience.*

**- Unsupported claims – the addition of the notes on color blindness needs to be supported with evidence (e.g. % of the sample that were colorblind and a reference to what this figure is being compared to).**

We added references as well as an explanation:

*“2% of the sample was colour blind and 60% of the declared wearing glasses or contact lenses to correct their vision, similar to the percentage in the UK population (34,35)^[1]^ .”*

*“2% of participants were colour blind and 57% wore glasses or lenses in Study 2.A while 1.5% were colour blind and 60% wore glasses or lenses in Study 2.B.”*

*34.	NHS. "Colour vision deficiency (colour blindness). 2017. Disponible sur: nhs.uk*

*35.	The College of Optometrists. Britain’s Eye Health in Focus. 2013*

**- row 132 ->: It is not clear how detectability was determined here, as the authors don’t explain what the pilot study participants actually did and this information comes later in the paper.**

We added the following for clarification:

*“This allowed us to calculate a measure of detectability d', which corresponds to participants' ability to detect the bin (see section “Analysis” below for a more detailed explanation) for each photo in the baseline condition and pick the 50 photos exhibiting bin discriminabilities detectability that were neither too low nor too high.”*

**- Ensure past tense is used for reporting the studies (e.g. Study 1)**

We thank the reviewer for pointing out this inconsistency. This is now corrected.

**- row 147: “We also collected INFORMATION ON socio-demographic variables”**

We have added the missing word.

**- row 269 ->: It would be useful if the authors provided further justification for testing additional colors, as this was provided in their response to the reviewers but not in the revised paper.**

Further explanation was provided for the use of the additional colors:

*“We applied the same tools and methodology to other colors most frequently considered in the visual saliency literature, blue and green (34,35). These two colors also bear practical relevance for the current study, as they have been considered by the Parisian municipality as potential alternative trash bag colors. In fact, green bags were used by the city from 2013 up until 2019 and were then replaced by grey bags for aesthetic reasons”.*

*34. Mullen KT, Kingdom F a. A. Differential distributions of red–green and blue–yellow cone opponency across the visual field. Visual Neuroscience. janv 2002;19(1):109‑18.*

*35.	Kamkar S, Moghaddam HA, Lashgari R. Early Visual Processing of Feature Saliency Tasks: A Review of Psychophysical Experiments. Frontiers in Systems Neuroscience.*

We would also like to draw the reviewer’s attention to the following footnote that had been added to the previous revised manuscript:

*“In the context of this study, our exchanges with the Parisian local authorities confirmed that the red, green and blue bags were provided by the same suppliers and cost the same as grey bags, so if the authorities decided to change their bags from grey to red, the cost would be zero. This, however, does not necessarily hold in all contexts where the production of a new coloured bag might incur a relatively low, but non-zero cost.”*

**- Sentence starting on row 217 should be revised for clarity**

We thank the reviewer for their comment. The sentence is now:

*“We therefore focused on the discriminability d', a metric that allows us to capture earlier stages of visual processing such as the sensory encoding of stimuli . d' represents the strength of the signal relative to noise, which corresponds to participants' ability to detect the bin”.*

**- sometimes discriminability is referred to as d´ and sometimes d0 – be consistent**

The notations are now consistent.

**- row 289: could be clearer what this means**

The sentence has been changed to:

*“Participants were pre-screened to have a 95% or above approval rating (i.e., participants with more than 95% of previous submissions on the platform being approved)”.*

**- row 296: is missing “308 for Study 2.B”**

We thank the reviewer for pointing out the typo.

**- check numbers – sample size varies between 305 and 300 for Study 2.A.**

We thank the reviewer for pointing out the typo.

**- Check consistency of incentives (between the studies they vary in currency ($ vs. €) and in value (7.50-62).**

We thank the reviewer for pointing this out. We would like to clarify that the studies were conducted on different platforms (to recruit French participants Study 4 was conducted on a French platform, which pays participants in euros). Moreover, Study 3 included only one question and required a survey time of less than 3 minutes, which explains the high fare (61 euros) per hour.

We have transformed all values to euros for the sake of consistency.

**- Row 339: explain what you mean by a “random sample”**

Following reviewer 4’s advice, we removed Study 3 from the current paper.

**- Row 345: please also summarize the demographic profile of this sample**

We would like to clarify that the survey administered in this study consisted only of one question. There was no collection of socio-demographic variables.

**- Row 388 – incomplete bracket**

**- rows 451 – 455: The same sentence is written twice.**

We thank the reviewer for pointing out these errors. We have corrected them in the manuscript.

**Reviewer #3: The manuscript is significantly improved in clarity and completeness of detail. I thank the authors for taking into consideration my previous comments, the changes made adequately respond to the majority of them. Nevertheless, I would like further clarification on one point. From my previous review:**

**“Although the dependent variable is the detection accuracy for individual bins in cityscape images, the authors refer several times in the text to the perceived density of bins. Since the latter is never measured directly or indirectly, I think the authors should revisit the claim that changing the colour of the bag produces an increase in the perceived density of the bins. If the authors assume a linear relationship between detection accuracy and the perceived density of the bins in the visual scene, they should at least substantiate this relationship with other data or with support from previous literature, since they only have one bin per image in this experiment. Perceived density may in fact depend not only on detectability, but also on salience and other contextual factors. The same applies to the conversion between the increase in detection accuracy and the absolute number of bins in the city and consequently also to the equivalent economic savings.”**

**As an answer to this concern in the body of the text you now report:**

***“Assuming a linear relationship, and for illustration, we speculate that a change of bag colour from grey to red, green or blue in the city of Paris could be equivalent to installing up to 8,370, 9,537 or 13,600 additional bins, respectively.”***

**Which is admissible in my opinion. However, In the abstract the sentence at lines 20 – 22 the sentence is formulated in a different way, leading to the impression that this is a result of the study.**

**“..assuming a linear increase in detection, we illustrate that this change could be equivalent to installing up to 8,370 bins in the city of Paris, in terms of perceived density.”**

Following the Reviewer’s comment, this section was removed altogether.

**Also at lines 106 – 110:**

**“We then convert the effect to a measure of perceived bin density and approximate the equivalent number of real bins that would have to be added in the streets of Paris to achieve the same effect..”**

**The calculation of the “the equivalent number of real bins” still puzzles me, because in some contexts it is presented as a result of the study when in my opinion it is speculative reasoning.**

**The linear function for the conversion here is just assumed (as you state at line 410). There is no evidence for the fact that this relationship should be linear, not even support from previous literature. Changing the colour of the bag as demonstrated in your study increases bin detection when the observer 1) is actively looking for the bin in the images 2) the image is presented for a limited amount of time. In most real-life situations, the time to visually scan the environment is not limited to a few milliseconds. When time pressure is low or absent, the advantage given by colour (or another feature that attracts attention in a bottom-up manner) will be smaller much smaller than then what found in your study. The speed accuracy trade-off in visual search is a well-known phenomenon, with reduced time pressure the accuracy will increase also for the hard serial scanning and the advantage given by bottom-up cues will be reduced. The increase in single bin detection you found in your images cannot therefore be linearly translated into perceived bin density in an ecological context where the time constraint is much less stringent.**

**Consequently, the equivalence between the increase in detection at your task and the number of bins installed under ecological conditions is purely speculative. Unless you have a way to substantiate (with data or citations) the existence of a linear relationship between single bin detection in your experiment and perceived bin density under real-life conditions, I would ask you to rephrase or remove from the text all sentences (including those in the abstract) related to the claim of equivalence between perceived density and the number of bins that would need to be installed to achieve a similar effect such as on line 313 (similar to the addition of 13,600 new bins).**

We removed the hit rate calculation and equivalent number of bins from the analysis, and incorporated the ROC area calculations proposed by Reviewer 4.

*“To measure the change in performance, we estimated the area under the received operating characteristic (ROC) of each colour condition as follows (Macmillan, 1993):*

$Ad'=\Phi(\frac{d'}{\sqrt{2}})$ *, where* $\Phi($*.) corresponds to the cumulative normal distribution. The ROC area can be interpreted as the proportion of times subjects would correctly identify the signal (38).Thus, an ROC area of 0.6 implies that signals would be correctly identified on 60% of the trials. ROC area calculations show that a participant was 6% more likely to correctly identify a bin with the red bag (87.8%) compared to the grey bag 82.8%).”*

*“ROC area calculations show that a participant was 6% more likely to correctly identify a bin with the green bag (Study 2.A: 88.9%, 83.8%) and 8.4% more likely to identify a bin with a blue bag (Study 2.B: 90.5%, 83.5%).”*

*“ROC area calculations show that a participant was 8.9% more likely to correctly identify a bin with the blue bag (92.5%, 84.9%) compared to a bin with a grey bag.”*

**Reviewer #4: This is an interesting article that merits publication, provided appropriate changes are made. In particular, additional details are needed regarding the participants, as well as the data analysis and findings. Furthermore, some of the conclusions go beyond what can be supported by the findings.**

**More information should be provided regarding the participants who were included and excluded. Participants were retained if they responded in at least 70% of the trials (and met other inclusion criteria), but a 30% missing data rate seems quite high. What was the average rate of missing data? Could an argument be made for treating the trials with no response as misses (when a trash bag was present) or correct rejections (when no bag was present)? How might this have affected the d' values?**

The way the experiment was designed does not allow participants to move on to the next trial without answering, so there are no missing answers. Missing values were generated when we removed trials for which reaction times were too short (150 milliseconds or less) or too long (4,000 milliseconds or more). This generated missing values that were taken into account when considering the 30% missing value threshold. This threshold was pre-registered so we decided to stick with our pre-registration plan. Please note that the average rate for missing data was 1% for Study 1 (no participant excluded), 1% for Study 2.A (1 participant excluded), 0.6% for Study 2.B (1 participant excluded) and 1% for Study 4 (1 participant excluded) .

The following histogram shows the occurrence of too fast/too slow answers, ie. the generated missing trials per individual, in Study 1. This pattern is similar for Studies 2 and 3.

**Studies 1 and 2 had far more females than males, while Study 4 showed the opposite trend. Were these gender imbalances the result of excluding more participants from one gender than the other, or did they simply reflect differences in the rates at which the two genders volunteered to participate?**

While Studies 1 and 2 were run on the Prolific Academic Platform and involved solely UK nationals, Study 4 was conducted on the Platform CrowdPanel, in order to access participants living in the Paris area. This might explain the discrepancy in the results since they simply reflect differences in the rates at which the two genders volunteered (67% female for Study 1, 58% for study 2.A, 65% for Study 2.B and 40% for Study 4) to participate and not the exclusion rate (which was not different according to gender).

**The authors chose not to exclude participants who described themselves as having color blindness (which should be described in the paper as "anomalous color vision). They claimed this would increase the generalizability of the findings, but males are much more likely to have anomalous color vision than females. Combining this with the gender imbalance of the studies means that ignoring gender and self-reported anomalous color vision in the analyses can actually reduce generalizability. The solution would be to include self-reported anomalous color vision as a covariate (although many people are not aware they have anomalous color vision), or testing the data for gender differences.**

We found no impact of gender in Studies 1, 2a and 2b. The following table shows the results of the three linear regressions regressing d’ on condition (colour vs. grey), gender and the interaction between both variables. The absence of significant effect of the interaction coefficient shows that the impact of the colour change was not different for men compared to women:

The table was added to the Supplementary Materials document and the following sentence was added in the results sections:

*“The result was not impacted by gender differences (see regression tables in Supplementary Materials).”*

**The manuscript provides very few descriptive statistics. Perhaps these could be provided in the OSF repository. In particular, hit and false alarm rates for each condition should be reported. Furthermore, d' values can only be calculated when hit and false alarm rates are greater than 0% and less than 100%. Were rates of 0% or 100% obtained for some participants in some conditions? If so, how often? What corrections were used to calculate d'?**

|  |  | Outcome rate | | |
| --- | --- | --- | --- | --- |
|  |  | Hit | | False Alarm |
|  | Occurrence of 0 false alarm rate or hit rate (corrections) | Grey | Colour |  |
| Study 1 | 13 | 0.51 | 0.61 | 0.13 |
| Study 2.A | 8 | 0.50 | 0.62 | 0.12 |
| Study 2.B | 5 | 0.52 | 0.68 | 0.12 |
| Study 3 | 8 | 0.55 | 0.73 | 0.13 |

Only some rates of 0 were obtained for a small number of participants in each case (13 in Study 1, 8 in Study 2.A, 5 participants in 2.B and 8 for Study 3). No participant remaining in the study had a false alarm rate of 100%. The instances of 0 were changed to 0.0001 in order to be able to calculate d’ for these individuals.

This table was also added to the Supplementary Materials as well as the following Summary statistics of our samples:

| **Table 2 Descriptive statistics, Study 1** | | | | |
| --- | --- | --- | --- | --- |
|  | **Overall, N = 308** |  | **Female, N = 204** | **Male, N = 104** |
|  |  |  |  |  |
| **Uses vision correction** | 60% |  | 70% | 40% |
|  |  |  |  |  |
| **Colour blind** | 1.6% |  | 0.5% | 3.8% |
| **Education** |  |  |  |  |
| Some high school or less | 1.0% |  | 1.0% | 1.0% |
| Completed high school | 15% |  | 12% | 21% |
| Some college | 19% |  | 20% | 15% |
| Completed college | 38% |  | 40% | 34% |
| Post graduate | 25% |  | 25% | 27% |
| Trade/technical school | 2.3% |  | 2.5% | 1.9% |
| **Age** Mean(SD) | 37(13) |  | 38(13) | 35(13) |
| **Discriminability**    ***d’ red*** | 1.65(0.72) |  | 1.60(0.72) | 1.75(0.70) |
| ***d’ grey*** | 1.34(0.62) |  | 1.28(0.64) | 1.46(0.57) |
|  |  |  |  |  |

| **Table 3 Descriptive statistics, Study 2.A** | |  | | |  | | |
| --- | --- | --- | --- | --- | --- | --- | --- |
|  | **Overall, N = 301** | |  |  | | **Female, N = 174** | **Male, N = 127** |
|  |  | |  |  | |  |  |
| **Uses vision correction** | 57% | |  |  | | 66% | 46% |
| **Colour blind** | 2.3% | |  |  | | 0.6% | 4.7% |
| **Education** |  | |  |  | |  |  |
| Some high school or less | 0.7% | |  |  | | 1.1% | 0% |
| Completed high school | 14% | |  |  | | 13% | 14% |
| Some college | 19% | |  |  | | 18% | 20% |
| Completed college | 40% | |  |  | | 40% | 39% |
| Post graduate | 26% | |  |  | | 26% | 25% |
| Trade/technical school | 1.0% | |  |  | | 0.6% | 1.6% |
|  | 0% | |  |  | | 0% | 0% |
| **Age** Mean(SD) | 36(12) | |  |  | | 36(12) | 36(12) |
| **Discriminability**  ***d’ green*** | 1.73(0.70) | |  |  | | 1.75(0.70) | 1.71(0.70) |
| ***d’ grey*** | 1.40(0.59) | |  |  | | 1.39(0.61) | 1.42(0.57) |
|  |  |  |  |  |  |  |  |

| **Table 4 Descriptive statistics, Study 2.B** | | | | |
| --- | --- | --- | --- | --- |
|  | **Overall, N = 308** |  | **Female, N = 201** | **Male, N = 107** |
|  |  |  |  |  |
| **Uses vision correction** | 60% |  | 65% | 51% |
| **Colour blind** | 1.6% |  | 0% | 4.7% |
| **Education level** |  |  |  |  |
| Some high school or less | 2.3% |  | 2.5% | 1.9% |
| Completed high school | 13% |  | 16% | 7.5% |
| Some college | 14% |  | 11% | 21% |
| Completed college | 42% |  | 41% | 43% |
| Post graduate | 28% |  | 30% | 23% |
| Trade/technical school | 1.3% |  | 0% | 3.7% |
| **Age** Mean(SD) | 37(12) |  | 38(12) | 35(12) |
| **Discriminability**  ***d’ blue*** | 1.86(0.71) |  | 1.80(0.69) | 1.97(0.73) |
| ***d’ grey*** | 1.38(0.63) |  | 1.34(0.63) | 1.47(0.61) |

| **Table 5 Descriptive statistics, Study 3** | | | | |
| --- | --- | --- | --- | --- |
|  | **Overall, N = 283** |  | **Female, N = 123** | **Male, N = 160** |
|  |  |  |  |  |
| **Uses vision correction** | 57% |  | 62% | 54% |
| **Colour blind** | 2.1% |  | 0.8% | 3.1% |
| **Age** Mean(SD) | 38(14) |  | 38(13) | 38(14) |
| **Discriminability**  ***d’ blue*** | 2.04(0.85) |  | 2.02(0.85) | 2.06(0.85) |
| ***d’ grey*** | 1.46(0.75) |  | 1.42(0.76) | 1.50(0.74) |
|  | | | | |

**In Study 1, the false alarm rate for calculating the grey bag d' is the same as the false alarm rate for calculating the red bag d'. Because of this, when the paired t-test was conducted, the false alarm rate drops out and the t-test is based entirely on the grey and red hit rates:**

**d' difference = z(Red hit rate) - z(FA) - [z(Grey hit rate) - z(FA)] = z(Red hit rate) - z(Grey hit rate)**

**This points to one of the issues in extrapolating from the d' values to the equivalent number of trash bins: The false alarm rates are ignored entirely in that calculation.**

**Furthermore, d' is a nonlinear variable that is difficult for most people (including policy makers) to understand. There is certainly tremendous appeal in an alternative metric that is more easily understood, such as the equivalent number of trash bins. However, it is a huge leap to jump from changes in d' to equivalent numbers of trash bins (and to jump from increases in the number of trash bins to decreases in littering, as previous reviewers have noted). It might be better to convert the d' values into estimated areas under the received operating characteristic (ROC). Think of the ROC area in relation to a task in which participants see two photographs. One contains a trash bag (of the color under study) and the other does not. The participant's task is to identify the picture that contains the trash bag. If the trash bag is easy to see (as in the catch trials) performance will be 100%. If the trash bag is virtually impossible to see the participant must guess and performance will be at the level of chance: 50%. To convert d' into RC area estimates, use the formula provided by Macmillan (1993) in Keren and Lewis (Eds.), A handbook for data analysis in the behavioral sciences: Methodological issues:**

**ROC area = inverse-z[ d' ÷ sqrt(2) ]**

**This formula predicts an ROC area of 87.8% for the red bags in Study 1 and 82.8% for the grey bags. Both bags have reasonably good saliency; performance is well above the chance level of 50%. Performance is 6% better with the red bag than with the grey bag. That may indeed be a notable increase in saliency, but it's a major leap to conclude that this is equivalent to a 28% increase in the number of trash bins. That simply wasn't tested.**

We removed the hit rate calculation and equivalent number of bins from the analysis, and incorporated the ROC area calculations recommended by the Reviewer.

*“To measure the change in performance, we estimated the area under the received operating characteristic (ROC) of each colour condition as follows (Macmillan, 1993):*

$Ad'=\Phi(\frac{d'}{\sqrt{2}})$ *, where* $\Phi($*.) corresponds to the cumulative normal distribution. The ROC area can be interpreted as the proportion of times subjects would correctly identify the signal (38).Thus, an ROC area of 0.6 implies that signals would be correctly identified on 60% of the trials. ROC area calculations show that a participant was 6% more likely to correctly identify a bin with the red bag ( 87.8%) compared to the grey bag 82.8%).”*

*“ROC area calculations show that a participant was 6% more likely to correctly identify a bin with the green bag (Study 2.A: 88.9%, 83.8%) and 8.4% more likely to identify a bin with a blue bag (Study 2.B: 90.5%, 83.5%).”*

*“ROC area calculations show that a participant was 8.9% more likely to correctly identify a bin with the blue bag (92.5%, 84.9%) compared to a bin with a grey bag.”*

**Study 3 seems very much out of place and isn't even mentioned in the Abstract. If it is retained (which I do not recommend) it needs to cite literature on how policy makers reach decisions -- and there needs to be a discussion explaining why the results from the participants (who are probably not policy makers by profession) can be generalized to actual policy makers**.

We removed Study 3 from the current paper and rephrased the discussion:

*“Indeed, if a policy maker wants to increase the perceived density of bins, it would be inefficient to simply rely on intuition or even previous laboratory research in different settings; for example, we show that even though red is reported most salient in a number of vision studies (27,28), it was not as good as blue in increasing the visibility of bins in the Parisian urban setting. It would also be inefficient to go straight to a costly field trial comparing various bin colour. Therefore, determining which colour is most likely to be salient in a particular city in an experimental setting first can therefore be a useful evidence-based first step before elaborating a field strategy”.*

**The authors have responded to many of the comments made by previous reviewers. However, there is surprisingly little mention of the need to conduct a field study to validate the findings. It's one thing to present photographs on a computer screen to participants who are instructed to find trash bins in those photographs; it may be entirely different to have people in natural settings decide what they're going to do with their litter. The studies reported by the authors are a good start, but there needs to be better recognition of their limitations.**

We expanded our limitation section and mentioned the need for field studies:

*“First, our set of experimental online studies may lack ecological validity and looking for a bin in real settings may rely on different mechanisms. Specifically, the short display time in our experiment mimics the impact of increased visibility when people are not actively looking for a bin, which differs from what happens in real-life. Therefore, although the results of our study provided initial evidence on the superiority of some colours compared to others in increasing salience, a field experiment is needed in order to validate the findings. Moreover, our studies do not allow us to state whether changing bag colour would have an effect on real-life littering. However, prior research provides suggestive evidence that making bins more attractive or noticeable affects behavior (20–22).”*

**One minor point concerns the descriptions of the participants in Study 2. The second set of statistics presumably applies to Study 2.B, but this is not indicated in the text.**

We thank the reviewer for pointing out this typo.

1. The lower number of colour blind rate (2% in the sample versus 4% in the population) reflects the overrepresentation of women in our sample: according to the NHS, 8% of men and 0.5% of women are affected by colour blindness in the UK population.
