# Supplementary material for: A zero-cost attention-based approach to promote cleaner streets: A Signal Detection Theory approach in Parisian streets
Source: PLoS One. 2023 Apr 26;18(4):e0284272. doi: 10.1371/journal.pone.0284272 (PMC10132669; doi:10.1371/journal.pone.0284272)
Supplement: Attachment — Submitted filename: Response to Reviewers.docx [file pone.0284272.s004.docx]

Reviewers' comments

Dear editor,

Thank you for giving us the opportunity to submit a revised draft of our manuscript. We appreciate the time and effort that you and the reviewers have dedicated to providing your feedback on our manuscript. We have incorporated the reviewers’ suggestions and highlighted the changes in red within the manuscript.

You will find below a point-by-point response to the reviewers’ comments.

**Reviewer #2: The authors have put considerable effort into revising the manuscript “A zero-cost attention-based approach to promote cleaner streets: a Signal Detection Theory approach in Parisian streets” and addressing my previous comments and concerns. The conducted research is now reported in a more concise and clear manner, and elaboration around relevant background literature (e.g. nudging) as well as study limitations have been included. Therefore, the rationale as well as practical implications of the research are now presented in a more sound manner, and overall the authors have made a stronger case for the relevance and significance of their research to the field of environmental psychology. However, some questions still remain surrounding the statistics, and some minor points regarding written expression have been listed in my comments below. Therefore I have recommended minor revisions.**

**Whilst this has improved significantly, the main area that still needs work is the reporting of the statistics. First, there is confusion on the chosen post-hoc analysis for Study 2a and b (row 330 onwards). From my understanding, after doing a 3x2 ANOVA, they examined the interaction further by running 3 separate 2x2 ANOVAS. I’m not convinced this has achieved their goal and still leaves questions on where the significant interaction lies. I’d recommend doing Simple Effects Analysis, e.g. comparing the three studies for the grey condition – so not statistically testing an interaction, but looking at a main effect of study but just on the grey condition (based on Fig 4, I suspect there will not be any differences) and then again for the 3 colours (of which I suspect some clear effects). This would lead to a much clearer and simpler narration of your findings (i.e. the studies found participants responded the same for the grey conditions, but there was a difference in colour…).**

We agree that adding a simple effect analysis clarifies the nature of the interaction. The results of the simple effects analysis were thus added in the results section:

*“An analysis of simple effects showed that the treatment effect was significant for the colour condition (between the different colours), F (2,912) = 6.40, p < 0.01, but not in the grey condition F (2, 912) = 0.73, p = 0.48. This suggests that participants responded the same for the grey condition across the three studies, but there was a difference in the coloured trial conditions.”*

However, we maintain the original two by two analysis because it is important to demonstrate that some colours are more superior compared to grey than others.

**The initial ANOVA was also not fully reported. As well as reporting the interaction, it is important to also report the main effects (e.g. study and colour).**

We thank the reviewer for pointing this out. The main effects were added to the results of the mixed ANOVA in the text:

*“The main effect for COLOUR was significant, F (1, 912) = 1191.40, p < .01,* $\eta^{2}=$ *.57, but the he main effect for STUDY was not significant, F (2, 912) = 2.73, p = .07,* $\eta^{2}=$ *.01.”*

**Second, the reporting of the ANOVA for Study 3 is unclear and needs further revision. E.g. the interaction and main effects need to be spelled out fully (row 381-382).**

We agree with the reviewer that the ANOVA reporting was unclear in the text. We added the following:

*“A mixed model ANOVA testing 2-level COLOUR factor (grey vs colour) x 2-level STUDY factor (study 2.B vs study 3) confirms that the effect of changing the colour from grey to blue was greater when considering a sample of residents in the Paris Area compared to a British sample; the interaction between STUDY and COLOUR was significant F(1,589) = 14.91, p < .001,* $\eta^{2}$*= .025. The main effects were also significant; STUDY F(1,588) = 5.07, p = .02,* $\eta^{2}$*= .01, COLOUR F(1,588) = 1585.63, p < .00,* $\eta^{2}$*= .73.”*

**Third and finally, the effect sizes need to be checked. This recent addition does make the paper stronger and clearer for the reader to follow, but the values are questionable. In the footnote, it implies Cohen’s dz is being used yet the text just notes d (and not dz). This needs to be explicit. For example, why is Cohen’s d not appropriate here (of which, should be under the value of 1)? If sticking with Cohen’s dz, the results are incorrect. I copied the footnotes’ formula, but did not replicate all of the reported findings. This needs to be checked.**

When comparing dependent means, the correlation between the observations has to be taken into account, and the effect size directly related to the statistical significance of the test (and thus used in power analysis) is Cohen’s dz (see Lakens, 2013). We agree that this should have been stated more clearly.

Footnote 2 was changed to:

“*The standardized mean difference effect size for within-subjects designs is referred to as Cohen's dz and its formula is based on calculations by Rosenthal (1991):*$d_{z}= \frac{t}{\sqrt{n}},$*, given the direct relationship between the t-value of a paired-samples t-test and Cohen's dz*”

Similarly, all instances of *“d”* were changed to *“dz.*

Lakens, D. (2013). Calculating and reporting effect sizes to facilitate cumulative science: a practical primer for t-tests and ANOVAs. Frontiers in Psychology, 4, 1-12. [863].

**In terms of minor points:**

**- Abstract, row 17: would be useful to clarify “trash can detection rates”.**

This sentence has been changed.

**- Row 56 & 60 -> unsupported claim needing a reference (even is this is not publically available, a personal correspondence reference is needed).**

The following reference has been added:

16.	France3 Paris. Paris : nouveau design pour les poubelles parisiennes. France 3 Paris Ile-de-France [Internet]. 2013 Nov [cited 3 nov 2022]. Available from: https://france3-regions.francetvinfo.fr/paris-ile-de-france/paris/paris-nouveau-design-pour-les-poubelles-parisiennes-366167.html

**- row 65 ->: The authors write: “we ask whether an increase in the visual saliency of bins can work as a substitute to adding bins”, but they have not compared these two strategies. I suggest rewording this part for clarity.**

We agree with the reviewer that this sentence was not very clear. It has thus been changed to:

*“In this study, we ask whether an increase in the visual saliency of bins can increase the perceived density of bins in a city.”*

**- row 68 ->: references to “a number of studies” are needed.**

The following references were added to that sentence:

19.	Arnold M. The relationship between receptacle design, normative conduct, environmental concerns, and recycling behavior. :37.

23	Geller ES, Brasted WS, Mann MF. Waste Receptacle Designs as Interventions for Litter Control. Journal of Environmental Systems. 1 janv 1979;9(2):145‑60.

24.	O’Neill GW, Blanck LS, Joyner MA. The use of stimulus control over littering in a natural setting. J Appl Behav Anal. 1980;13(2):379‑81

**- Row 189 -> unsupported claim needing a reference**

The following reference was added:

35.	Afrin S, Rahman A, Islam F, Hoque F. Environmental effects of tourism. 2013;1(7):15.

**- Row 199 -> the numbers still do not add up (203 females + 104 males = 307, not 308). As noted in the previous review, if there is a respondent who didn’t state their gender, this needs to be explicit.**

This typo was corrected.

**- Row 259-260 -> unsupported claim needing a reference**

The following reference was added:

43.	Wong AL, Haith AM, Krakauer JW. Motor planning. Neuroscientist 21: 385–398, 2015. doi:10.1177/1073858414541484.

**- row 262 ->: explanation of how ROC was estimated and why could go in the Method section.**

We thank the reviewer for this suggestion. The following sentence was added at the end of the methods section:

*“Then, to measure the change in performance in a more intuitive manner, we estimated the area under the received operating characteristic (ROC) of each colour condition using the* $d^{'}$ *as follows (Macmillan, 1993) (38):* $A_{d^{'}}= \phi\left( \frac{d^{'}}{\sqrt{2}} \right)$*, where* $\Phi\left( \cdot\right)$ *corresponds to the cumulative normal distribution. The ROC area can be interpreted as the proportion of times subjects would correctly identify the signal (39). For example, if the trash bag is easy to see, ROC area will be equal to 1. If the trash bag is virtually impossible to detect the participant must guess and the ROC area will be at level of chance: 0.5.”*

**- Row 297 -> could state “As applied in Study 1, trails for which…” to emphasise continuity between studies.**
**- Rows 318-319 -> be consistent in decimal places (e.g. should the SDs have .00 added to them to be in-line with the M units?)
- Row 351 -> “replicated the study” – this is confusing as three studies have previously been reported. This could be rephrased to “replicated the design as used in the previous studies…”**
**- Throughout -> as above, the manuscript often refers to “the Study” (e.g. row 65 & 396) yet 3 studies are reported. Rephrase (e.g. “this paper”?)
- Throughout -> ensure spelling is consistent (E.g. US spelling – color; or UK spelling – colour)**

All of these revisions have been addressed.

**Reviewer #4: This submission addresses the concerns I had regarding previous versions. I have only a few suggested changes, all of them minor.**

**The participants were asked whether or not they had colour blindness, but the proper term is anomalous color vision. I would suggest using the term anomalous colour vision, and explaining (in parentheses) that participants were asked if they had colour blindness. This applies to Lines 203 and 302, and the footnote on page 15.**

The instances of “colour blindness” in the text was changed to “anomalous colour vision” as suggested.

**In Lines 267-268, readers might confuse the explanatory statistics (ROC area of 0.6) with the study findings (ROC areas of 87.8% and 82.8%). I suggest rephrasing the text using the actual study findings. For example, for the red bag the ROC area of .878 indicates that participants would were 87.8% accurate in identifying the presence of red bags.**

We agree that the wording was confusing to the reader. The sentence now reads:

*“ROC area calculations show that participants were accurately identified the bin in 87.8% of the trials (*$A_{d_{red}^{'}}=$*0.878) and 82.8% accurate in identifying the presence of a grey bag* $(A_{d_{grey}^{'}}=$*0.828). Participants were thus 5% more likely to correctly identify a bin with the red bag compared to the grey one”.*

**There are a few minor mathematical inconsistencies that should be addressed (unless they are due to rounding issues). On Line 268, the two ROC areas differ by 5% rather than 6%. On Line 376, the d’ for blue bags appears to be 40% higher than for grey, not 39%. On Line 383, the ROC areas appear to be 9.0% higher for blue than for grey, not 8.9%.**

The rounding inconsistency was corrected.

**In the Discussion, very briefly discuss the possibility of policy makers having concerns about increasing the saliency of the bags, especially in areas of Paris that target tourists. It may be that a conscious decision was made to select an “invisible” bag color so as not detract from the visual surroundings.**

The following paragraph was added in the discussion.

*“While increasing the saliency of bins can decrease littering, it might entail esthetic costs. This is especially problematic in touristic cities with architectural heritage, like Paris. To help policy makers make informed decisions facing this trade-off, more evidence on the benefits of more visible bins is needed”.*

**In the Supplementary material, add information about the number of participants excluded for each reason in each study.**

**We thank the reviewer, and we agree that this information needs to be provided. We add the following table to the Supplementary materials.**

**Table 6 Participant exclusion rates**

|  |  | **Initial N** | **Failed 5 or more catch trials** | **Unrealistic Reaction time on more than 30% of trials** | **Final N** |  |
| --- | --- | --- | --- | --- | --- | --- |
| **Study 1 (red)** |  | 324 | 16 | 0 | 307 | |
| **Study 2.A (green)** |  | 312 | 11 | 0 | 301 | |
| **Study 2.B (blue)** |  | 315 | 7 | 0 | 308 | |
| **Study 3 (blue – French sample)** |  | 307 | 24 | 1 | 282 | |

**Finally, there are some minor typographic corrections. In Line 204, “of the declared” should read “of them declared.” On Line 255, round the mean to 1114 (to maintain same level of precision as the mean reaction time for red bags).**

This typo was corrected
