# Supplementary material for: A zero-cost attention-based approach to promote cleaner streets: A Signal Detection Theory approach in Parisian streets
Source: PLoS One. 2023 Apr 26;18(4):e0284272. doi: 10.1371/journal.pone.0284272 (PMC10132669; doi:10.1371/journal.pone.0284272)
Supplement: Attachment — Submitted filename: Response to Reviewers.docx [file pone.0284272.s005.docx]

Response to Reviewers

We thank the reviewers for examining the article in detail and for providing insightful comments. We have addressed all the minor points pointed out in this review. Please see details below.

The minor points (row numbers refer to the tracked changes document):

Introduction:
- rows 47 – 48 : “increase in the number of bins might increase litter in the presence of litter in the environment” -> Rewording suggested for clarity.

*Done*

- Rows 95-97: update to a more relevant example or remove

*Done*

All Method sections
- Provide a brief justification of the target sample size. Each study had a very specific sample size, considering the researchers paid for this amount, an explicit rationale is needed in why they asked Prolific for each sample size
*Done*

All Results sections
- Ensure formatting of the results is consistent throughout the paper (e.g. the letters should be italicised, but the numbers not, p value should be reported fully unless < .001 [row 335+]; all decimals should be the same e.g. to 2 decimal places)

*Done*

- Provide a recap summary of the main take home finding(s) for each study before moving on to the next section. Just needs to be 1-2 sentences.

*We thank the reviewer for this suggestion. We have added the following summaries after each results section:*

*“In summary, bins with red bags are more visible than bins with grey bags.”*

*“In summary, Study 2’s results of show that changing the colour of bags from grey to green and from grey to blue significantly increases their visibility. A comparison between the three colour conditions shows that a bin with a blue bag is significantly more visible than one with red or green bag.”*

*“In summary, we observe the same results when we consider a Parisian (local) sample; the visibility of a bin increases when its bag is blue compared to grey. Study 3 also shows that the change in visibility is greater for Parisian participants than it is for British participants.”*

Methods (Study 1):

- rows 258 – 259: “The difference between mean discriminability in the two colour conditions” should be rephrased as “The difference in mean discriminability between the two colour conditions” for clarity.

*Done*

Methods (Study 2):
- rows 324-327: statements need to be supported with reference(s).

*Since this observation resulted from personal exchanges with employees of the Parisian munucupality, the following reference has been added:*

*“(Personal communication, the department of cleanliness and sanitation (DPE) of the Paris municipality, 2021).”*

Results (Study 2):
- row 371 onwards: When reporting the results from the mixed model ANOVA, the dependent variable should be stated.

*Done*

- rows 399 – 401: I would suggest moving this part about the Study 2 -specific ROC results before the mixed ANOVA. In this way all Study 2 -specific results are grouped, ensuring a more logical flow.

*Done*

- row 425: can omit “also”.

*Done*

Methods (Study 3):
- No colour blindness rates reported for this sample. Could state that this data could not be collected.

*We thank the reviewer for pointing this out. The following sentence has been added:*

*“2% of the sample reported anomalous colour vision (participants were asked whether they were colour blind) and 61% of them declared wearing glasses or contact lenses to correct their vision.”*

Results (Study 3):
- e.g row 436 onwards: Should clearly state what the dependent variable was, e.g. “… the effect of changing the colour from grey to blue on d’ was greater when…”.

*Done*

- row 471 onwards: “increasing the saliency of bins can decrease littering might entail esthetic costs…” should be reworded for clarity (also esthetic -> aesthetic).

*Done*

- rows 494 – 495: “In all these cases, the bin is at the same distance in the eye-catching condition and in the control condition but littering rates are different.” -> Not clear whether this refers to Studies 1 – 3 or to the existing studies reviewed just before.

*Done*

- row 508: Should be “colours”.

*Done*

- row 508: Omit “Therefore” to avoid repetition.

*Done*

Reviewer #4: The use of italics on lines 338 and 348-351 differs from the use of italics elsewhere in the manuscript. Also, line 429 is missing a word -- or perhaps "can" should be replaced with "to."

*Done*
